# Structural characterization of a pathogenicity-related superoxide dismutase codified by a probably essential gene in *Xanthomonas citri* subsp. *citri*

**DOI:** 10.1371/journal.pone.0209988

**Published:** 2019-01-07

**Authors:** Diego Antonio Leonardo Cabrejos, André Vessoni Alexandrino, Camila Malvessi Pereira, Deborah Cezar Mendonça, Humberto D'Muniz Pereira, Maria Teresa Marques Novo-Mansur, Richard Charles Garratt, Leandro Seiji Goto

**Affiliations:** 1 Laboratório de Biologia Estrutural, Grupo de Cristalografia, Instituto de Física de São Carlos, Universidade de São Paulo, São Carlos, SP, Brazil; 2 Laboratório de Bioquímica e Biologia Molecular Aplicada—LBBMA, Departamento de Genética e Evolução, Universidade Federal de São Carlos, São Carlos, SP, Brazil; Instituto Butantan, BRAZIL

## Abstract

Citrus canker is a plant disease caused by the bacteria *Xanthomonas citri* subsp. *citri* that affects all domestic varieties of citrus. Some annotated genes from the *X*. *citri* subsp. *citri* genome are assigned to an interesting class named "pathogenicity, virulence and adaptation". Amongst these is *sodM*, which encodes for the gene product XcSOD, one of four superoxide dismutase homologs predicted from the genome. SODs are widespread enzymes that play roles in the oxidative stress response, catalyzing the degradation of the deleterious superoxide radical. In *Xanthomonas*, SOD has been associated with pathogenesis as a counter measure against the plant defense response. In this work we initially present the 1.8 Å crystal structure of XcSOD, a manganese containing superoxide dismutase from *Xanthomonas citri* subsp. *citri*. The structure bears all the hallmarks of a dimeric member of the MnSOD family, including the conserved hydrogen-bonding network residues. Despite the apparent gene redundancy, several attempts to obtain a *sodM* deletion mutant were unsuccessful, suggesting the encoded protein to be essential for bacterial survival. This intriguing observation led us to extend our structural studies to the remaining three SOD homologs, for which comparative models were built. The models imply that *X*. *citri* subsp. *citri* produces an iron-containing SOD which is unlikely to be catalytically active along with two conventional Cu,ZnSODs. Although the latter are expected to possess catalytic activity, we propose they may not be able to replace XcSOD for reasons such as distinct subcellular compartmentalization or differential gene expression in pathogenicity-inducing conditions.

## Introduction

*Xanthomonas citri* subsp. *citri* is a Gram-negative, rod-shaped, flagellated bacterium, known to be the causative agent of citrus canker (Asiatic canker A), one of the most widespread and destructive diseases of citrus groves. Citrus canker affects all of the domesticated citrus varieties known to date, and continues uncontrolled in citrus cultivating areas. The disease causes brownish corky wounds, frequently surrounded by a yellow halo due to an overall reduction of pigmentation in all parts of the plant, including the leaves and fruit. Infected fruit lose both quality, due to the extent of the lesions, and productivity, due to premature fruit fall. Nevertheless, fruit fall is not a consequence of the lesions themselves, but is a systemic symptom caused by abnormal production of phytohormones. There is no effective treatment nor prevention for the disease and its mitigation is mostly achieved by elimination of affected plants. Transmission occurs by simple contact, either by natural agents or by human plant management [1].

Efforts to better understand the infection mechanism, with a view to developing a more effective treatment include genomic and proteomic studies [2–9] as a means to identify key molecules or mechanisms which could be used as targets for the control of the disease. Structural biology [10–11], and random [12–13] or targeted mutations [14–16] are also techniques which have been used for gene-to-disease studies.

Found within a gene class designated "pathogenicity, virulence and adaptation" in the annotated genome of *Xanthomonas citri* subsp. *citri* strain 306 is the *sodM* (XAC2386) gene, which encodes a predicted superoxide dismutase (XcSOD) [2]. Local proteomics studies showed that *X*. *citri* subsp. *citri* SOD may present an increase in abundance when the bacteria are grown in the pathogenicity-inducing XAM-M medium, as compared to Nutrient Broth (NB) [9], implying its involvement in the disease process.

Superoxide, one of the so-called Reactive Oxygen Species (ROS), normally results from incomplete O_2_ reduction during aerobic metabolism and can damage many different biomolecules if not promptly neutralized [17]. In plants, ROS are actively used to eliminate infective microorganisms [18]. The rapid accumulation of ROS after pathogen recognition, commonly referred to as the oxidative burst, has been implicated in both direct antimicrobial defense as well as in cellular signaling leading to the induction of plant defense gene expression [19]. Indeed, an extracellular SOD from *X*. *campestris* has been shown to be an elicitor of the induced oxidative burst in plants [20].

Superoxide dismutases are metalo-oxidoreductases (EC: 1.15.1.1) that catalyze the dismutation of the superoxide radical (O2^•-^) into molecular oxygen and hydrogen peroxide (H_2_O_2_). They generally act in concert with catalases that further dismute H_2_O_2_ into innocuous oxygen and water [21]. During the dismutation reaction the substrate acts by alternating as an electron donor and acceptor, supported by the metal ion. Depending on the redox metal cofactor, SODs can be classified into three main structural groups: Cu,ZnSODs which contain both metals, MnSOD/FeSODs which have either one or the other metal, and the NiSODs [22].

In this work, molecular cloning was used as the first step towards characterizing the product of the *sodM* gene (XAC2386), predicted to be a *X*. *citri* subsp. *citri* SOD, with the objective of performing functional and structural studies on the recombinant protein (XcSOD). These were complemented by attempts to obtain a deletion mutant, by double homologous recombination, as a means to acquire additional information concerning the influence of XcSOD on infection. Although the *X*. *citri* annotated genome shows three additional sequences related to SODs [2], a deleted *sodM* mutant could not be obtained, indicating that this SOD gene could be essential for bacterial survival and that the remaining SOD genes are unable to compensate for its absence. Heterologous expression of *sodM* provided pure, homogeneous, catalytically active XcSOD, from which a high resolution structure was determined by X-ray crystallography. Homology modeling was used in order to explain why the three remaining *X*. *citri* SOD genes, *sod*C2, *yoj*M and *sod*B (XAC0210, XAC0209 and XAC2677, respectively) are unable to compensate for XcSOD deficiency.

## Materials and methods

### General procedures

*Xanthomonas citri* subsp. *citri* 306 was stored as -80°C reminders in 20% v/v glycerol stocks. Custom oligonucleotides were provided by IDT. Restriction enzymes, PCR reagents and cloning vectors were all from Fermentas. pNPTS138 was kindly supplied by Prof. Dr. Henrique Ferreira (UNESP, Rio Claro, SP, Brazil). Additional reagents were of analytical grade. General molecular biology techniques used throughout this work were as previously described [23].

### Cloning and construction of expression and deletion vectors

Isolation of DNA fragments was done by PCR using *X*. *citri* genomic DNA as template. Similar to previous reports [10, 16], for recombinant expression, the XcSOD coding sequence was amplified using oligonucleotides designed to incorporate an *Nde*I restriction site (underlined) at the 5’ end of the PCR product (5’ TACCCATATGGCTTACACCCTTCCGCAGTTGC) and a *Xho*I site at the 3’ extremity (5' TATATATACTCGAGTCAGGCGATCGCGGCGTGG), immediately after the XAC2386 predicted stop codon. For construction of the gene deletion vector, two distinct 1 kb regions adjacent to the SOD structural sequence were independently amplified. For the upstream region, *Hind*III and *BamH*I restriction sites were adapted to the 5' (5' AAGCTTGTGCTGGCCAGCGCCTGGG) and 3' (5' GGATCCGCGTATCTCCTGGACTTGCCGCC) ends respectively. For the downstream region, *Bam*HI (5' GGATCCGGTGCTGCATCGGCGCTAGG) and *Nhe*I (5' GCTAGCCAAGAGTGGCCGCTACACCTGG) sites were employed. PCR was performed using a C1000 Touch (Biorad) thermal cycler programmed to execute an initial 7 min denaturing step at 97° C, followed by 35 cycles of 94° C for 30 s, 60° C for 30 s, 72° C for 1.5 min, and a final elongation step of 10 min at 72° C. Each fragment was amplified using 100 ng of genomic DNA and 100 pMol of each oligonucleotide in defined pair combinations in 50 μL reactions. Amplification products were gel-purified, cloned into pJET 1.2 (Fermentas) and transformed into *E*. *coli* DH5α for propagation. All the plasmid inserts were completely confirmed by sequencing [24] on a 3130 Genetic Analyzer (Applyed Biosystems).

DNA encoding XcSOD was excised using *Nde*I and *Xho*I restriction enzymes and subcloned into pET28a (Novagen). The constructed plasmid provides IPTG induced expression of XcSOD fused to an *N-*terminal His-tag.

The 1 kb *sodM* flanking regions were excised from the cloning vectors using their respective adapted restriction sites (*Hind*III-*Bam*HI and *Bam*HI-*Nhe*I) and sequentially subcloned into the respective sites of pNPTS138 (Alley Dickon, unpublished results). The constructed deletion vector carries a kan^R^ selection mark and also provides sucrose suicide selection [25]. The deletion vector was used to transform cells of wild type *X*. *citri* subsp. *citri* (0.2 cm gap cuvette, 2.5 kV pulse, 200 Ω resistance and 25 μF capacitance) and transformants were selected on LB agar containing kanamycin at 30 μg/mL. Aiming to obtain plasmid-free double crossing-over *sodM* deleted mutants from these, isolated transformants were grown for 24 h on plain LB broth. Subsequently, 0.2 μL of the bacterial cultures were spread over LB agar containing 10% sucrose, and as the constructed vector carries a *sacB* suicide marker, surviving colonies must have lost the plasmid containing the deleted gene. Survivors should comprise both unchanged cells and the expected deletant which could be differentiated by PCR.

### Recombinant expression and protein purification

The expression vector was transformed into *E*. *coli* BL21 (DE3) (Novagen) and expression was carried out in an orbital shaker at 250 rpm, 18°C in LB broth containing 30 μg/mL kanamycin for 16 h after the addition of 0.1 mM IPTG to the culture during mid-log growth. Cells from 1 L culture were collected by centrifugation and re-suspended in 50 mL of 25 mM Tris-HCl pH 8.0 100 mM NaCl. Cell lysis was performed by ultrasound pulses in an ice bath and insoluble cellular debris was removed by centrifugation. Lysate soluble extract was loaded onto a 5 mL Ni-NTA column (Novagen) pre-equilibrated with the same buffer. For IMAC purification, the column with bound protein was initially washed with 50 mL of 2 mM imidazole in the same buffer and purified XcSOD was eluted in 10 column volumes of 250 mM imidazole in the same buffer. Imidazole was removed by dialysis against 25 mM Tris-HCl pH 8.0 100 mM NaCl and XcSOD was stored at -20°C and subsequently used for enzyme activity measurements. Aliquots from every XcSOD preparation were reserved for SDS-PAGE analysis [26] where the 203 residue XcSOD monomer was expected to appear with a molecular mass of 22.7 kDa. Estimates of the XcSOD concentration were made from the absorbance at 280 nm using a calculated [27] extinction coefficient of 0.1% (g.L^-1^) = 1.932.

For the estimation of the oligomeric state of the native enzyme, size exclusion chromatography was employed. In this case cells were resuspended in lysis buffer containing 50 mM Tris-HCl pH 8.0, 150 mM NaCl, 0.6 mM PMSF and were treated with 50 mg/mL lysozyme for 30 mins followed by sonication. Affinity chromatography was performed as described above. The recombinant protein was treated with thrombin (1U/mL) at 4°C for two days in order to remove the affinity tag. Size exclusion chromatography was performed using a Superdex 200 10/300 column driven by an Akta purifier (GE Healthcare) in the same buffer excluding PMSF. The flow rate used was 1.5 mL/min with a pressure limit of 1.5 MPa and detection at 280 nm. The single protein peak corresponding to recombinant XcSOD was concentrated and stored for crystallization experiments.

### Enzyme activity assay

SOD activity was measured [28] using the Superoxide Dismutase Activity Assay Kit, ab65354 (Abcam). Samples were prepared according to the manufacturer´s instructions. Measurements were performed in an iMark (Biorad) microplate reader, using clear flat-bottom 96 well microtiter plates, recording the absorbance at 450 nm after 20 min incubation at 37°C with triplicated serial dilutions (0.0009–0.59 μM) of purified XcSOD.

### Protein crystallization and structure determination

XcSOD, in 50 mM Tris pH 8.0 150 mM NaCl, was prepared as described in section 2.3. Several different protein concentrations were tested during crystallization trials. Suitable XcSOD crystals were obtained in 0.1 M Bis Tris pH 6.1, 25% PEG 3350 employing a protein to buffer ratio of 1:2 μL using a protein concentration of 7.2 mg/mL. A suitable crystal was rapidly cryo-cooled in liquid nitrogen (20% PEG 200 added to the mother liquor) and diffraction data were collected to 1.8 Å resolution on a Rigaku MicroMax 007 HF / R-AXIS IV++ (Rigaku Co.) system, using radiation from a copper anode at the *Laboratório de Biologia Estrutural*, (IFSC—USP, São Carlos, SP, Brazil). The XDS Package [29] was used for data processing. The XcSOD structure was solved by molecular replacement using Phaser [30] employing the structure of superoxide dismutase from *Bacillus subtilis* (PDB ID 2RCV) [31], which shares 66% sequence identity, as the search model. The structure was refined in reciprocal space using phenix.refine [32] and the maps and models visualized with Coot [33]. R and R_free_ were used as the main criteria for validating the refinement protocol and Molprobity was used to evaluate stereochemical parameters of the model during refinement. [34]. Table 1 gives the data processing and model refinement statistics. The coordinates and structure factors for XcSOD have been deposited in the PDB (PDB ID: 6BEJ).

**Table 1:** Data processing and structure refinement statistics.

Data Collection	*Xc*SOD
Space Group	*P2*_*1*_ *2*_*1*_ *2*_*1*_
Cell dimensions	
*a*, *b*, *c (Å)*	60.67, 72.47, 87.67
Detector	Raxis-IV
Wavelength (Å)	1.5418
Resolution range (Å)	26.33–1.89 (1.96–1.89)
Multipliciy	
*R*meas (%)	6.2 (50.0)
CC(1/2)	99.9 (94.3)
Completeness(%)	98.26 (92.76)
Total reflections	172966 (25296)
Unique reflections	30876 (2861)
I / σ(I)	17.75 (3.57)
Refinement parameters	
Reflections used for refinement	30863 (2859)
*R* (%)	20.27
*R*_Free_(%)	25.10
No. of protein atoms	3154
No. of ligand atoms	2
Solvent	304
B (Å^2^)	38.14
Coordinate Error (ML based) (Å)	0.24
Phase error (^o^)	27.12
Ramachandran Plot	
Favored (%)	97.24
Allowed (%)	2.76
Outliers (%)	0.00
All-atom Clashscore	2.78
RMSD from ideal geometry	
r.m.s. bond lengths (Å)	0.004
r.m.s. bond angles (°)	0.69
PDB ID	6BEJ

### Molecular modeling

Homology models for the remaining SOD gene products of *Xanthomonas citri* were generated using the modeling software Modeller 9.18 [35]. Initially default Blastp searches [36] were used to confirm that XAC0210 and XAC0209, here called Xac1 and Xac2 respectively, belong to the Cu,ZnSOD family, while XAC2677, here called Xac3, is a MnSOD/FeSOD. Alignments for modeling purposes were generated with the program Clustal via the Jalview interface [37]. Xac1 and Xac2 models were built using a SOD-like mutant enzyme from *B*. *subtilis* (pdb code 1XTM) as template. Further sequence analysis using the characteristic residues described by Bleicher *et al*. [38] revealed that Xac3 was an iron-containing enzyme and was modeled based on its homologue from *E*. *coli* (1ISA). Equivalent atoms were transferred from the template to the model and the remainder positioned according to standard protein geometry prior to coordinate randomization in Cartesian space followed by ten model generation steps using VTFM [39]. Models were refined with "very slow" simulated annealing and ranked according to their DOPE scores. Further quality evaluation was performed with Procheck [40], Verify_3D [41] and the quality parameter of What_Check [42].

## Results

### The lack of mutants

As described previously [10, 16], screening trials for mutants were performed by PCR using an additional pair of oligonucleotides which are complementary to regions present in *X*. *citri* subsp. *citri* genomic DNA and which flank 50 bp before and after the region of interest located 1 kb upstream and 1 kb downstream, respectively. Thus, *sodM* deletants were expected to show a PCR product around 2 kb while unchanged wild type cells should be close to 2.6 kb. We have assessed more than 300 individual surviving colonies for one week and also surveyed several 25 μL batches from different grown transformants but no mutant with an expected PCR product was found (results not shown).

### Enzyme activity

**Fig 1:**
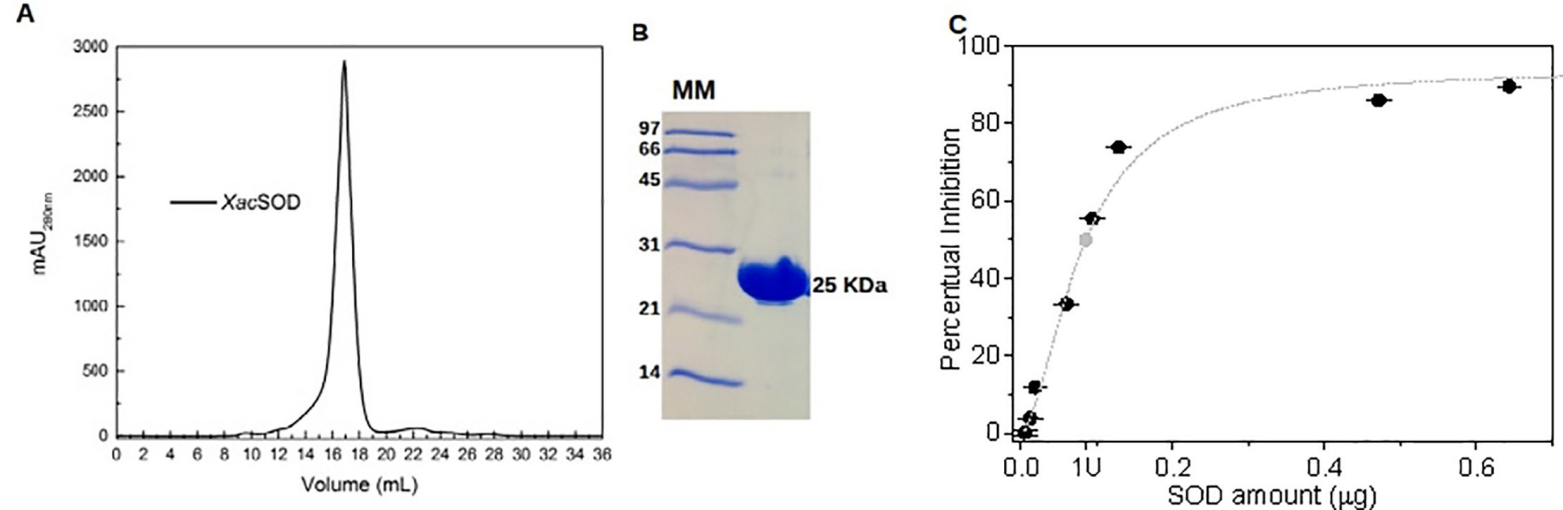
Purification and activity of XcSOD. (A) Size exclusion chromatography on Superdex 200 10/300 showing the native protein to be dimeric in solution, (B) SDS-PAGE indicating the purity of the final product and (C) concentration dependent enzyme activity measured as the percentage reduction in the formation of WST-1-formazan as a result of superoxide removal by XcSOD.

XcSOD was purified as a homodimer as estimated from its molecular mass using size exclusion chromatography (Fig 1A and 1B). XcSOD activity was estimated using a Superoxide Dismutase Activity Assay Kit ab65354 (Abcam). Briefly, the assay infers dismution of the superoxide anions normally produced by xanthine oxidase which can be measured by reduction of a sulfonated tetrazolium salt (WST-1) to a water-soluble WST-1-formazan that absorbs light at 450 nm. The increase in absorption, which is linearly related to the xanthine oxidase activity, is indicative of the rate of WST-1 reduction by superoxide anions which in turn can be dismuted by SOD. Therefore, the activity of SOD can be determined by the inhibition of color development and is expressed as the amount of enzyme needed to inhibit 50% of the xanthine oxidase production of superoxide anions [28]. One unit of XcSOD is thus defined as 8.614 x 10^−2^ μg of protein (Fig 1C).

### Protein structure

XcSOD crystallized in space group *P*2_1_2_1_2_1_ with two monomers per asymmetric unit. Crystals diffracted to high resolution and data were processed to 1.85 Å. Table 1 shows data collection, processing and refinement statistics together with standard stereochemical quality parameters, all within expected values.

**Fig 2:**
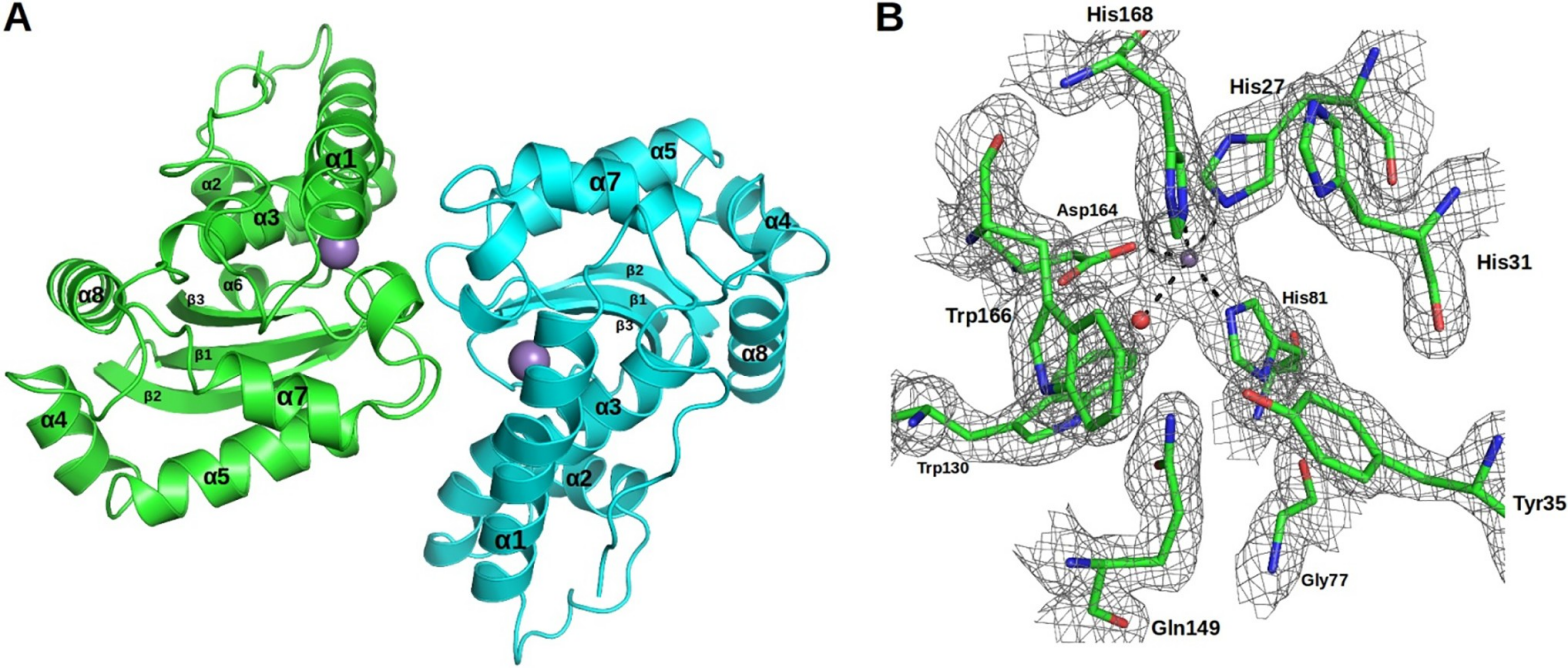
The crystal structure of XcSOD. (A) A canonical dimeric structure compatible with the sequence signatures described in the text. Secondary structure elements are labeled and the Mn^2+^ ions are indicated as spheres. (B) The active site of XcSOD showing the ligands to the metal ion and the corresponding electron density (2F_obs_-F_calc_ contoured at 1σ).

XcSOD shows the well-known fold characteristic of both MnSOD/FeSODs (Fig 2A) and presents an overall RMSD of 0.35 Å on Cα atoms when a monomer is superposed on its closest homologue, the MnSOD from *B*. *subtilis*, which was used for molecular replacement. On overlaying the dimer, the RMSD rises only slightly to 0.58 Å indicating that not only is the fold highly conserved but the relative orientation of the two monomers also. On extending these comparisons to other dimeric MnSODs of known crystal structure (twelve in total, whose sequence identities with XcSOD varied between 38 and 65%), the RMS deviations spanned a range from 0.43 to 0.83 Å, indicating that both the tertiary and quaternary structures of the family as a whole, vary very little. Furthermore, when the crystal structure of the Fe-substituted MnSOD from *E coli* is compared with XcSOD the RMSD is effectively no different to that observed for the unsubstituted enzyme, reinforcing the notion that the effect of metal substitution is subtle and local in nature. The quality of the final electron density map can be seen in Fig 2B which shows the active site region including the Mn ion.

**Fig 3:**
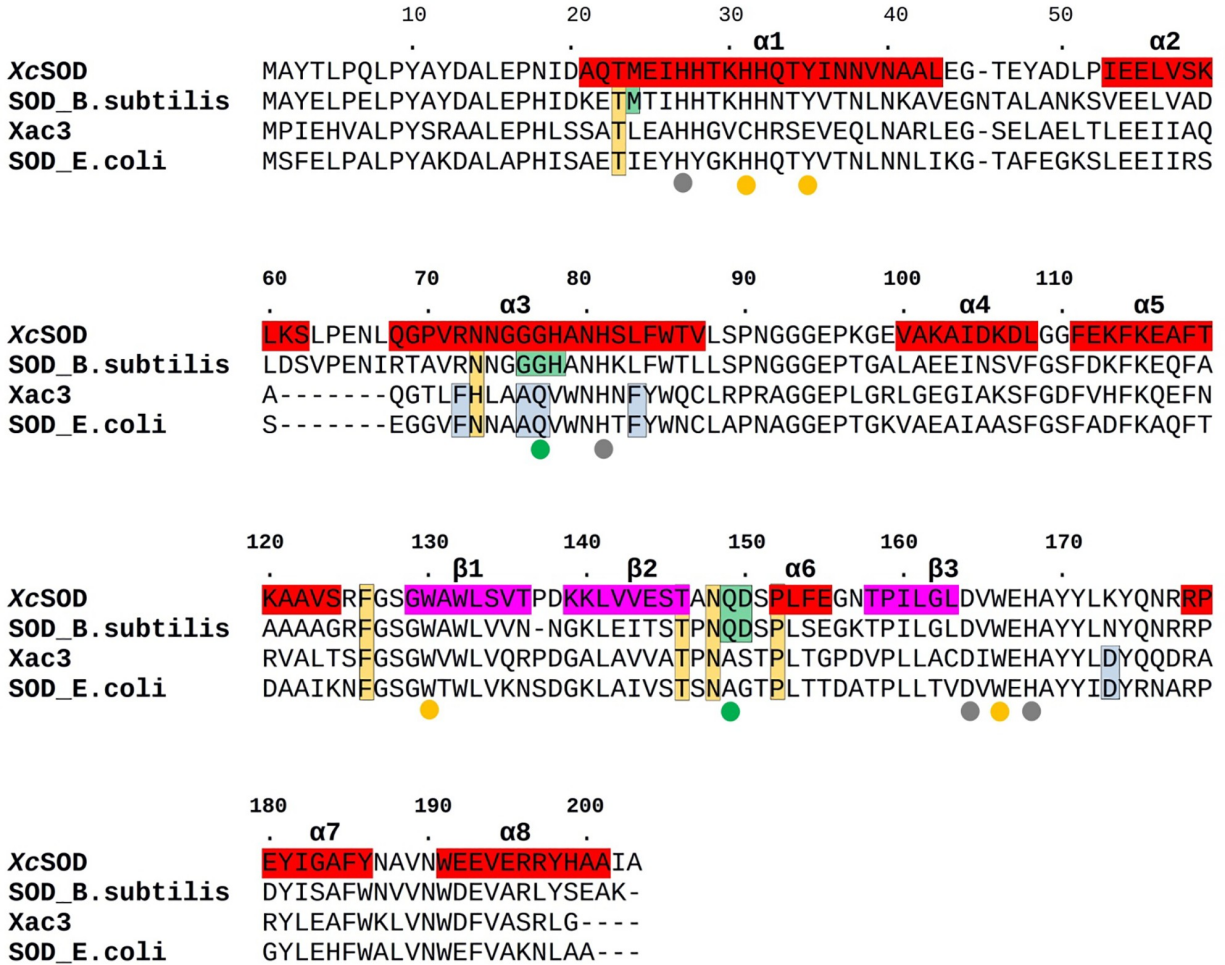
Alignment of MnSODs from *Xanthomonas citri* (XcSOD) and *Bacillus subtilis* together with the FeSOD from *E*. *coli* and Xac3. Secondary structure elements are highlighted in red (α-helices) and purple (β-strands). The colored dots indicate metal-binding residues (grey), active site vicinal residues (yellow) and the glutamine which donates a hydrogen bond to the ligand (green). The latter resides in a different position in the sequences for Mn and Fe bearing enzymes. Residues which are characteristic of either Mn-containing enzymes or Fe-containing enzymes are boxed with green or blue backgrounds respectively, whilst those characteristic of dimers are boxed in orange. These residues have been derived from previous alignment studies [24,25] and the *B*. *subtilis* and *E*. *coli* sequences presented here are used merely as representatives of the two groups of enzyme.

MnSOD/FeSODs can be either dimeric or tetrameric enzymes. In the case of XcSOD the two monomers in the asymmetric unit are related to one another in such a way as to present all of the hallmarks of a dimeric enzyme consistent with the SEC results (Fig 1A) and the crystallographic symmetry. These are most notably characterised by the lack of the large antiparallel α-helical hairpin composed of α1 and α3 which is typical of tetrameric enzymes. This is accompanied by the concomitant appearance of an additional helix (α2). These observations are entirely consistent with an analysis of the amino acid sequence of XcSOD, which has all of the residues characteristic of dimeric SODs, described by Bleicher *et al*. [38] based on previous work by Wintjens *et al*. [43], including Thr23, Asn73, Phe126, Thr146, Asn148 and Pro152 (Fig 3).

**Fig 4:**
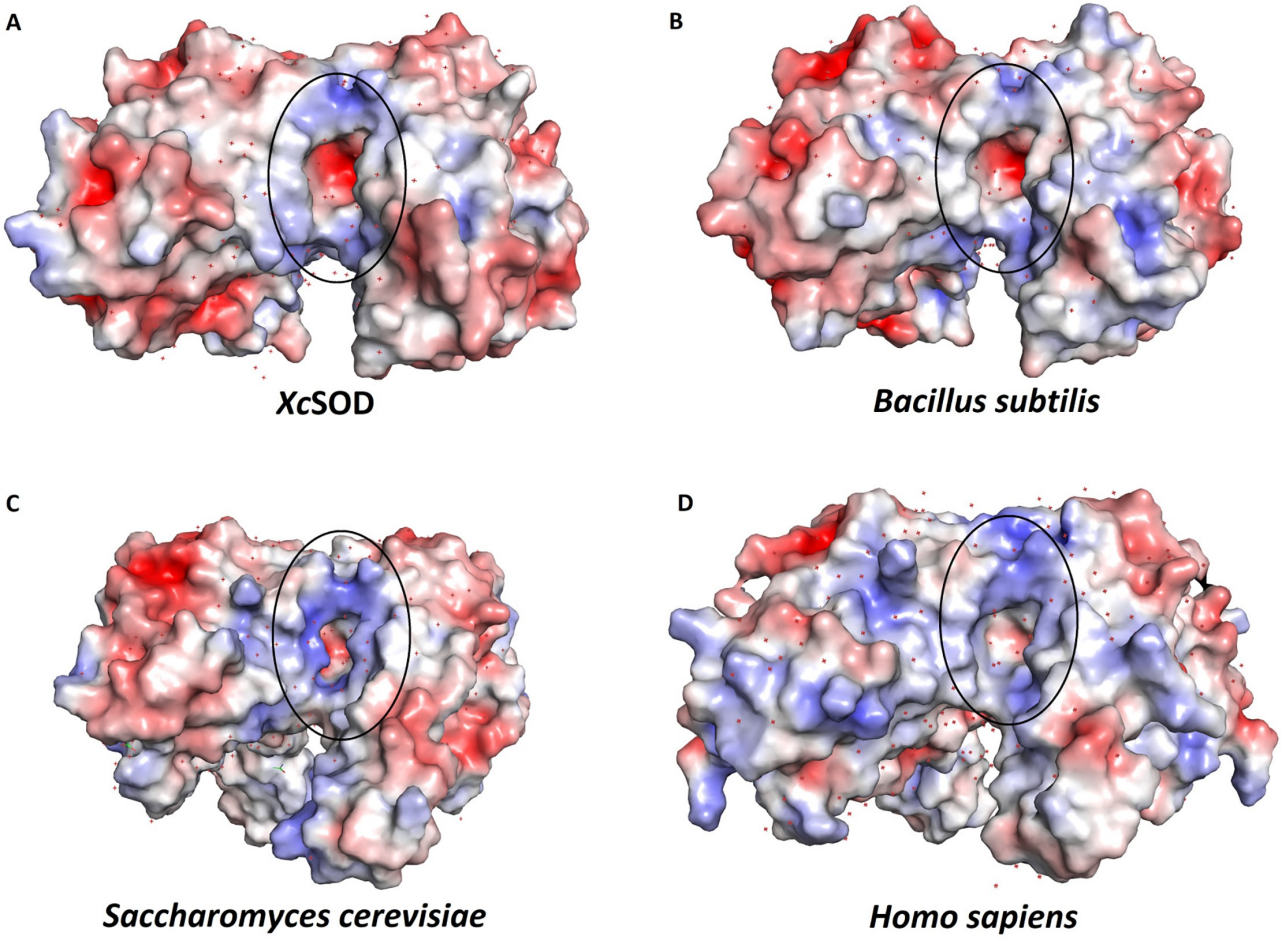
The electrostatics of the entrance channels of MnSODs. All electrostatic potentials have been coloured using the same scale so that they are strictly comparable. The enzymes from *X*. *citri* and *B*. *subtilis* are both dimeric whilst those from *S*. *cerevisiae* and *H*. *sapiens* are tetrameric. In the latter cases a dimer has been isolated from the tetramer for the purposes of calculating the electrostatic potential. The black oval highlights the ring of positive electrostatic potential around the active site. Although the details vary from structure to structure they all share this common feature.

Further analysis of the amino acid sequence also reveals that XcSOD has all of the residues previously identified to be fingerprints of a Mn-containing enzyme and therefore was refined as such. These residues are highlighted in Fig 3 and include Met24, Gly76, Gly77, His78, Gln149 and Asp150. The Mn ion is coordinated by Asp164, His81, His27, His168 and a solvent derived ligand (HOH304). Residues which are important for defining the topography and electrostatic potential of the access channel to the metal are highly conserved and include Trp130 and Trp166 as well as His31,Tyr35 and Gln149 which participate in the conserved network of hydrogen bonds necessary for securing the metal-bound solvent ligand or superoxide (S1 Fig). The electrostatic potential around the active site is very similar to that observed in other members of the FeSOD/MnSOD family and presents a halo of positive charge directing the substrate anion towards the active site. This is true for both dimeric and tetrameric MnSODs and is due to the conservation of residues equivalent to Lys30, His31, and Arg178 of XcSOD (Fig 4). These are the homologues of Lys37, His38 and Lys190 which perform a similar role in *S*. *cerevisiae* [44]. These authors also comment on the negative potential observed towards the bottom of the channel (also observed here for XcSOD) and we speculate that this may also play a role in efficiently locating the substrate at the active site. Charged residues which are not generally conserved within the family are concentrated in regions of the molecule which are not critical for substrate guidance (Fig 4). Allied to the fact that the hydrogen bonding network mentioned above is also fully conserved, these observations suggest that it is therefore reasonable to assume that XcSOD acts by the catalytic mechanism established for other Mn superoxide dismutases [22].

### Homology modeling

The homology models of Xac1, Xac2 and Xac3 were validated using three independent methods: Procheck [40] for standard stereochemistry, Verify_3D [41] for residue environments and What_Check [42] for atomic contacts. The Procheck G-factors in all cases were equivalent to those expected for crystal structures of 1.5 Å or better and had a minimum of 86% of residues within the most-favored regions of the Ramachandran plot and a maximum of one residue with a disallowed conformation. Values of 89%, 75% and 99% of all residues in the Xac1, Xac2 and Xac3 models respectively had 3D_1D scores above 0.2 according to the Verify_3D analysis. The course packing quality analysis of What_Check showed all three models to be of either good (Xac3) or normal (Xac1 and Xac2) quality. In summary, all validation techniques showed the models to be of reliable quality for subsequent analysis.

**Fig 5:**
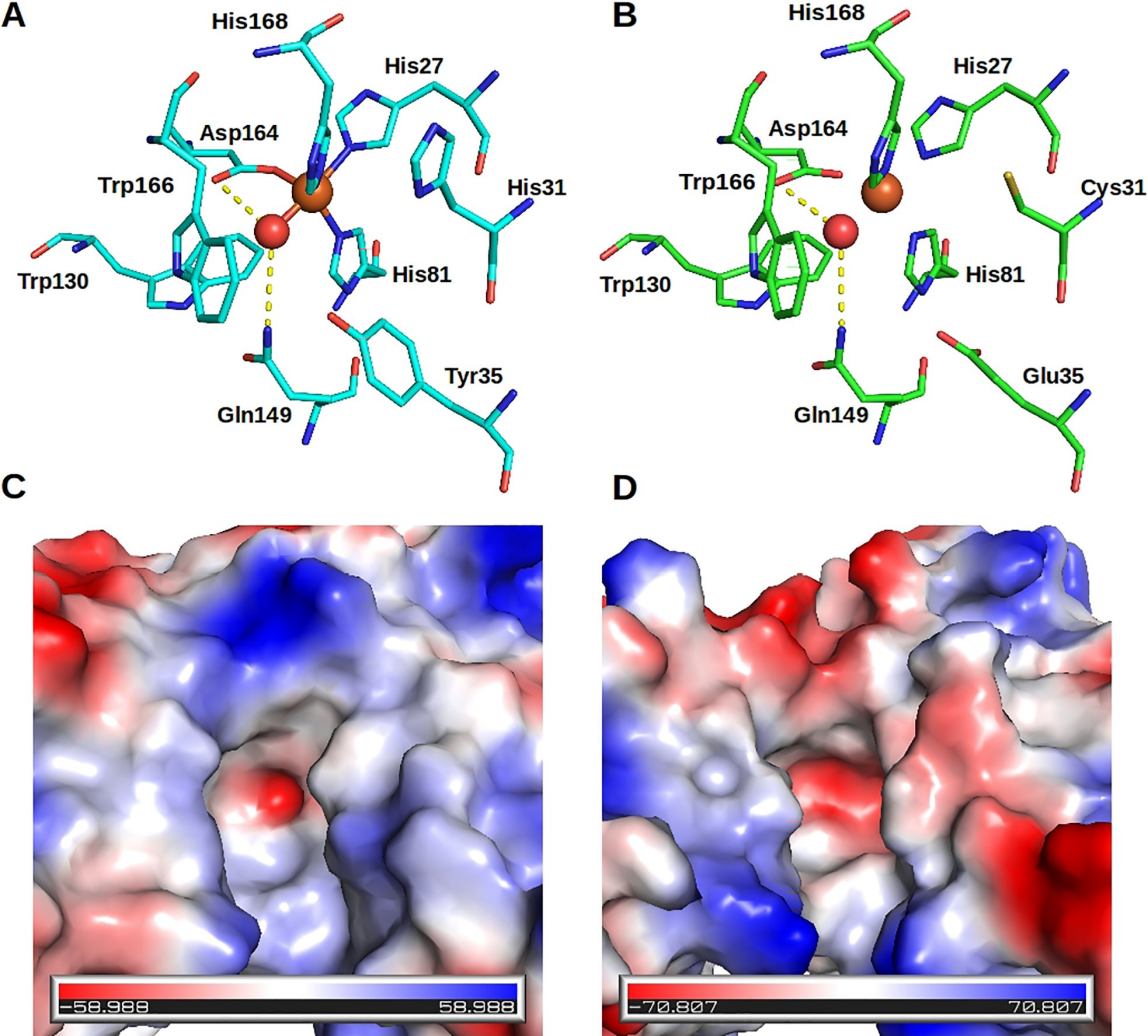
FeSOD active sites. (A) and (B) show the active sites of the FeSOD from *E*. *coli* and Xac3 respectively. The metal ion and its coordinated water-derived ligand are shown as orange and red spheres respectively. His31 and Tyr35 have been substituted by Cys and Glu respectively leading to dramatic changes to the chemical characteristics of the active site. These include the electrostatic potential which is more negative in the case of Xac3, disfavoring the approach of the incoming superoxide substrate (C and D).

In the case of Xac3, the sequence presents all of the hallmarks of a dimeric enzyme [38], with the exception of His73 (following the numbering of XcSOD as in Fig 3). Its specificity for iron as the metal cofactor can be inferred from the presence of Phe72, Ala76, Gln77, Phe83 and Asp173. However, significant differences are observed within the active site cavity due to the substitution of His31 in XcSOD by Cys and of Tyr35 by Glu. These substitutions change the topography and electrostatics of the channel leading to the active site (Fig 5). In particular Tyr35 is a highly conserved residue in both MnSODs and FeSODs which normally secures the orientation of the active site glutamine, essential for substrate binding.

The homology models of Xac1 and Xac2 were based on the crystal structure of a mutant of a SOD-like protein from *B*. *subtilis* (PDB file 1XTM). This protein is normally catalytically inactive due to a lack of two histidine residues which are ligands to the active site copper. The template shares approximately 30% sequence identity with both Xac1 and Xac2 and in the mutant structure used for modeling the two histidines have been reintroduced in order to restore catalytic activity (S2 Fig). The models reveal no significant structural variation in the vicinity of the active sites with respect to other Cu,ZnSODs. Although there is a deletion in the region of the electrostatic loop when compared with the bovine enzyme, the guiding arginine (Arg141 in the bovine SOD) is present and there are no significant differences to the electrostatic potential which guides the substrate towards the active site (S3 Fig).

## Discussion

*X*. *citri* subsp. *citri* has aerobic habits, inevitably leading to the generation of oxidative stress during normal respiration. Furthermore, as a pathogenic microorganism, it faces additional oxidative stress during plant infection [18, 45]. As a consequence, SOD genes are induced unusually quickly during the early stages of *Xanthomonas* colonization [46–47].

In *E*. *coli*, the regulation of the MnSOD encoded by *sod*A (also known as *sodM*) initially seems to be very complex being affected by heat shock [48], oxygen tension [49], O_2_^•-^ generating agents [50], iron chelators and is also induced by the presence of paraquat [51]. Furthermore, it responds to changes in the redox state of the cell [52]. *Sod*A is a member of the *soxRS* regulon which is activated by intracellular superoxide levels and the presence of MnSOD at high concentrations can act by lowering the expression of all the enzymes that are upregulated by intracellular levels of O_2_^•-^ including MnSOD itself [53]. Selenium metabolism can result in O_2_^•-^ formation and although inducing both Mn an Fe SODs, the mechanisms behind these are distinct [54]. Oxidative stress generated by paraquat or hydrogen peroxide treatment induces only MnSOD but not FeSOD, as opposed to iron excess in the surrounding environment, a condition that decreases MnSOD and increases FeSOD [55]. The induction of MnSOD and FeSOD are controlled by SoxR and Fur transcriptional regulators respectively [56]. On the other hand, Cu,ZnSOD encoded by s*odC* [57], which is not a member of *soxRS* regulon, is not induced by either cytosolic or periplasmic superoxide stress. The Cu,ZnSOD is delivered into the periplasmic space late during the stationary phase of cell culture. It seems to be induced by RpoS in aerobic conditions, and repressed by Fnr in anaerobic conditions [58]. In summary, of the these three kinds of SODs, MnSOD is the most responsive to intracellular superoxide levels or to conditions that interfere with these.

Since the negatively charged superoxide radical (O2^•-^) cannot simply cross the cell membrane [59], SODs would be expected to be specialized to specific cellular compartments. This has been observed for pathogenic bacteria affecting animals [60–61] as well as a freshwater nonpathogenic bacterium [62]. Indeed, a SodM was identified as one of the most abundant proteins in the extracellular proteome of *X*. *campestris* pv. *campestris*, which in turn activates defense mechanisms, called the oxidative burst, in the plant [20], indicating that SodM can be sensed by the host. Previously, our proteomics studies indicated a positive correlation between *X*. *citri sodM* expression and the canker infection [9], and a similar correlation was described for *X*. *campestris* [20, 47, 63] suggesting a significant role for SodM in the disease process. However, the unambiguous subcellular localization of XcSOD remains controversial. Whilst we observe SodM from *X*.*citri* in a periplasmic enriched fraction [9], in the case of *X*. *campestris* it was observed in both the extracellular and whole cell extracts, but not in the periplasm [64]. Nevertheless none of these homologues was predicted to reside outside the cytosol, as can be inferred from both the absence of a signal peptide and/orXX Cu,Zn metal cofactors. For instance, *E*. *coli* has all three different bacterial SODs which,according to their metal cofactors, function at different sub-cellular locations. Both FeSOD and MnSOD are present in the cytoplasm while Cu,ZnSOD is located in the periplasm. The FeSOD is attributed to play the house-keeping role because of its constitutive expression while the expression of MnSOD fluctuates in response to internal O_2_^•-^ levels, and the Cu,ZnSOD enzyme is present at minimal levels in the periplasmic space in order to protect membrane constituents from exogenous superoxide [65]. As a result, the apparent extracellular localization of XcSOD, an MnSOD, might be due to bacterial lysis or to an unknown mechanism, as has been commented on previouslyfor other bacterial SODs based onproteomic analyses where it was consistently identified in extracellular protein samples [64, 66].

*X*. *citri* is expected to use versatile antioxidant defense strategies, which are reflected in the presence of a great number of genes for antioxidant mechanisms: the SoxR and OxyR sensors, four predicted catalases and the four putative SODs [2, 67]. Thus, it seems reasonable that a "pathogenicity, virulence and adaptation" related SOD may work independently from similar housekeeping enzymes. Since we were unable to obtain a deleted *sodM* (XAC2386) mutant, this led to the suggestion that XcSOD may be an essential enzyme. Even though this seems initially unexpected, a similar lack of MnSOD mutants has already been described for *X*. *campestris* [47].

The presence of different SODs is a conserved feature within *Xanthomonas* as in some other bacteria. Indeed it is relatively common to find MnSOD, FeSOD and Cu,ZnSOD simultaneously present within a given species [68] as observed here. As expected, the essential SOD described by Smith *et al*. for *X*. *campestris* [47] and our XcSOD (XAC2386) belong to the very same group, the MnSODs, and have only a single difference in terms of amino acid sequence, suggesting very similar structures, regulation and roles. This could explain why, for both genes, mutation was equally unsustainable while for the three remaining SODs, it remains to be proven feasible or not. Despite our best efforts we were unable to find any report of a SOD mutant in any species of *Xanthomonas* while in other bacteria these have been widely described [69–72]. This potentially implies unique features of the *Xanthomonas* SOD system as a whole, which is worthy of further investigation.

With respect to catalysis, XcSOD presented a specific activity of 11609 U/mg, very similar to the value of 12800 U/mg reported for *Yersinia enterocolitica* SodA [73], a tetrameric MnSOD with which it shares 57% sequence identity. The dimeric MnSOD from *Bacillus subtilis*, used here for molecular replacement, presents a slightly lower activity but is nevertheless still of the same order of magnitude (4590 U/mg) [74]. Indeed different classes of SODs will often show similar efficiencies due to diffusion-limited catalysis. For example four FeSODs from the protozoan *Trypanosoma cruzi* showed specific activities ranging from 7380.89 to 13956.52 U/mg and a Cu,ZnSOD from *Cordyceps militaris* exhibited a specific activity of 27272.7 U/mg [75]. In summary the value reported here for XcSOD is within the expected activity range.

In order to gain further insight into the characteristics of the manganese containing XcSOD, our first approach was to determine its crystal structure and complement it with homology models for the products of the remaining three *X*. *citri* SOD genes (Xac1, Xac2 and Xac3). These provided us with the possibility of examining the full structural landscape for *Xanthomonas* SODs.

Initially, it is of interest to note that a homology built model has already been proposed for XcSOD itself using the SwissModel software [76]. The model was built on the basis of the *B*. *subtilis* enzyme used in the present study for molecular replacement. It is well known [77] that at 68% sequence identity reliable models can be readily produced, but it is still of interest to learn from mistakes made by automated software [78–79]. Fig 3 shows that there are only two indels on comparing the sequences of *X*. *citri* and *B*. *subtilis* MnSODs. One, around residue 45 represents a deletion of one residue in XcSOD and the model predicts a loop conformation which is completely incorrect (S4 Fig). The other, an insertion around residue 140, has been correctly modeled, including the prediction of a type I β-turn in the hairpin loop between β1 and β2 (not present in the template) complete with a main chain hydrogen bond (S4 Fig). Insertions have been traditionally considered more difficult to predict than deletions and it is interesting to note that the opposite proved to be the case here, possibly because of the predictability of the β-turn given the sequence [80]. Clearly, there is still much to be learned about the errors committed during homology modeling and there is an imperative for experimentally derived structures, such as that described here, if they are to form the basis of future experiments. More surprising was the result of the homology modeling for the predicted FeSOD (Xac3 or XAC2677) which presented two non-conservative substitutions within the active site pocket when compared to other Fe or Mn-containing enzymes. Tyr35 (XcSOD numbering) forms part of the hydrogen-bonding network necessary for permitting proton transfer during catalysis. It interacts with both Gln149 (which secures the metal-bound solvent or superoxide ion) and His31 (via a water molecule). Therefore, the substitution of both Tyr35 and His31in Xac3 destroys the absolutely conserved hydrogen-bonding network. The importance of Tyr35, His31 and the hydrogen bonding network as a whole has been borne out by many experiments using site directed mutagenesis [81–83]. Furthermore, the replacement of Tyr35 by a glutamic acid alters the charge distribution within the substrate cavity and would be expected to disfavor the approach of an incoming superoxide anion. On the other hand, the substitution of His31 by cysteine introduces a chemically reactive residue within the active site as cysteines are known to be readily oxidized by H_2_O_2_ or superoxides to sulfenic acid. In fact, in the case of GAPDH, such an alteration can even lead to the generation of a catalytically altered non-phosphorylating form of the enzyme [84–85].

Indeed, a search for sequence homologs of Xac3 revealed that *Xanthomonas* species in general have FeSODs which present the very same two mutations within the active site cavity. However, on excluding *Xantomonoadaceae* from the database search, no further examples were found and the closest homologs, from species of *Stenotrophomonas*, *Arenimonas*, *Lysobacter*, *Pseudomonas*, *Marinobacter* presented approximately 60% sequence identity with Xac3 and a conventional active site. These observations strongly suggest that the activity of the *Xanthomonas* FeSODs has been seriously compromised and may well be either inactive or have an unknown function. Furthermore, this appears to be a specific characteristic of *Xanthomonas sp*. Clearly, this requires some empirical validation beyond the scope of the current work but is consistent with the failure to retrieve MnSOD deletion mutants from *Xanthomonas*, something which has been successful in the case of other bacteria [69–70, 72].

As for the two remaining SOD genes of *X*. *citri*, XAC0210 and XAC0209, they code for Cu,ZnSODs (Xac1 and Xac2), which are evolutionarily distant from the MnSOD/FeSOD family. Sequence alignment (S2 Fig) shows that both Xac1 and Xac2 are lacking the DXT motif of the disulphide loop, common in eukaryotic Cu,ZnSODs and important for the formation of a dimeric interface. However, prokaryotic enzymes often present a completely different quaternary structure which utilizes an alternative dimerization surface. The residues characteristic of such an interface are also absent from both Xac1 and Xac2, indicating that these enzymes are probably monomeric, similar to that of *E*. *coli* [86]. The well conserved active site and neighboring electrostatic potential (S3 Fig) is highly suggestive that both Xac1 and Xac2 should be catalytically active.

The combination of our structural and genetic studies raise an important question: Why are the three remaining enzymes unable to substitute for MnSOD (XcSOD) in a deletion mutant? Firstly, it is highly likely that Xac3 is catalytically inactive. Secondly, the Cu/Zn enzymes may be restricted to specific functions, associated with the distinct subcellular localizations of different types of SOD in the bacteria [62], or with differential expression during growth or infective phases [46, 87]. For example, it is well known that MnSOD and FeSOD tend to be associated with the cytosol whereas Cu,ZnSODs are found in the periplasmic space [17]. Their roles might not necessarily be replaceable, or alternatively could be only partially overlapping. In the present case, with a presumed catalytically deficient cytosolic FeSOD (the product of the Xac3 gene), the other cytosolic SOD (XcSOD) for which we have here presented structure and activity data, might have become efficient enough and essential, for sole survival even in pathogenicity, virulence or adaptation conditions in *Xantomonoadaceae*.

## Supporting information

S1 Fig The conserved hydrogen bonding network observed in XcSOD. Residues around the active site which aid in securing the substrate superoxide anion are indicated.
(TIFF)

S2 Fig Alignment of the two Cu,ZnSODs from *Xanthomonas citri* (Xac1 and Xac2). Sequence alignment of Xac1 and Xac2 was done with the bovine enzyme (SOD_BOV) and sequences from *Photobacterium leiognathi* (Photo), which presents an alternative dimeric interface to that seen in the bovine enzyme, and that from the SOD-like protein mutant from *Bacillus subtilis* (1XTM) used for model construction. Elements of secondary structure are highlighted in purple (β-strands) and red (α-helix) and the DXT motif in light blue. Residues involved in the alternative dimeric interface of the *Photobacterium* SOD are highlighted in grey. Copper and zinc ligands, the disulphide cysteines and the active-site arginine (important for the second half-reaction), are indicated with blue, grey, yellow and red dots respectively.
(TIFF)

S3 Fig The electrostatic potential around the active sites of Cu,ZnSODs. (A) shows the canonical charge distribution observed in the bovine enzyme, (B) and (C) correspond to Xac1 and Xac2 respectively. Despite their differences, all three structures show a marked positive potential leading to the active site.
(TIFF)

S4 Fig Failures and successes of the Swissmodel homology model for XcSOD. (A) shows the vicinity of residue 45 where a deletion has been incorrectly modeled by the automatic software and (B) shows the correctly predicted structure for a one-residue insertion. In both cases the crystal structure is shown in green and the homology model in blue.
(TIFF)
