# Correction: Structural characterization of a pathogenicity-related superoxide dismutase codified by a probably essential gene in *Xanthomonas citri* subsp. *citri*

**DOI:** 10.1371/journal.pone.0231016

**Published:** 2020-03-23

**Authors:** Diego Antonio Leonardo Cabrejos, André Vessoni Alexandrino, Camila Malvessi Pereira, Deborah Cezar Mendonça, Humberto D’Muniz Pereira, Maria Teresa Marques Novo-Mansur, Richard Charles Garratt, Leandro Seiji Goto

The affiliation for the second author is incorrect. The correct affiliation is not indicated. André Vessoni Alexandrino is not affiliated with Laboratório de Bioquímica e Biologia Molecular Aplicada—LBBMA, Departamento de Genética e Evolução, Universidade Federal de São Carlos, São Carlos, SP, Brazil but with: Programa de Pós-Graduação em Biotecnologia, PPGBiotec, Universidade Federal de São Carlos, SP, Brazil.
