# Supplementary figures and images for: Structural characterization of a pathogenicity-related superoxide dismutase codified by a probably essential gene in Xanthomonas citri subsp. citri
Source: PLoS One. 2019 Jan 7;14(1):e0209988. doi: 10.1371/journal.pone.0209988 (PMC6322740; doi:10.1371/journal.pone.0209988)

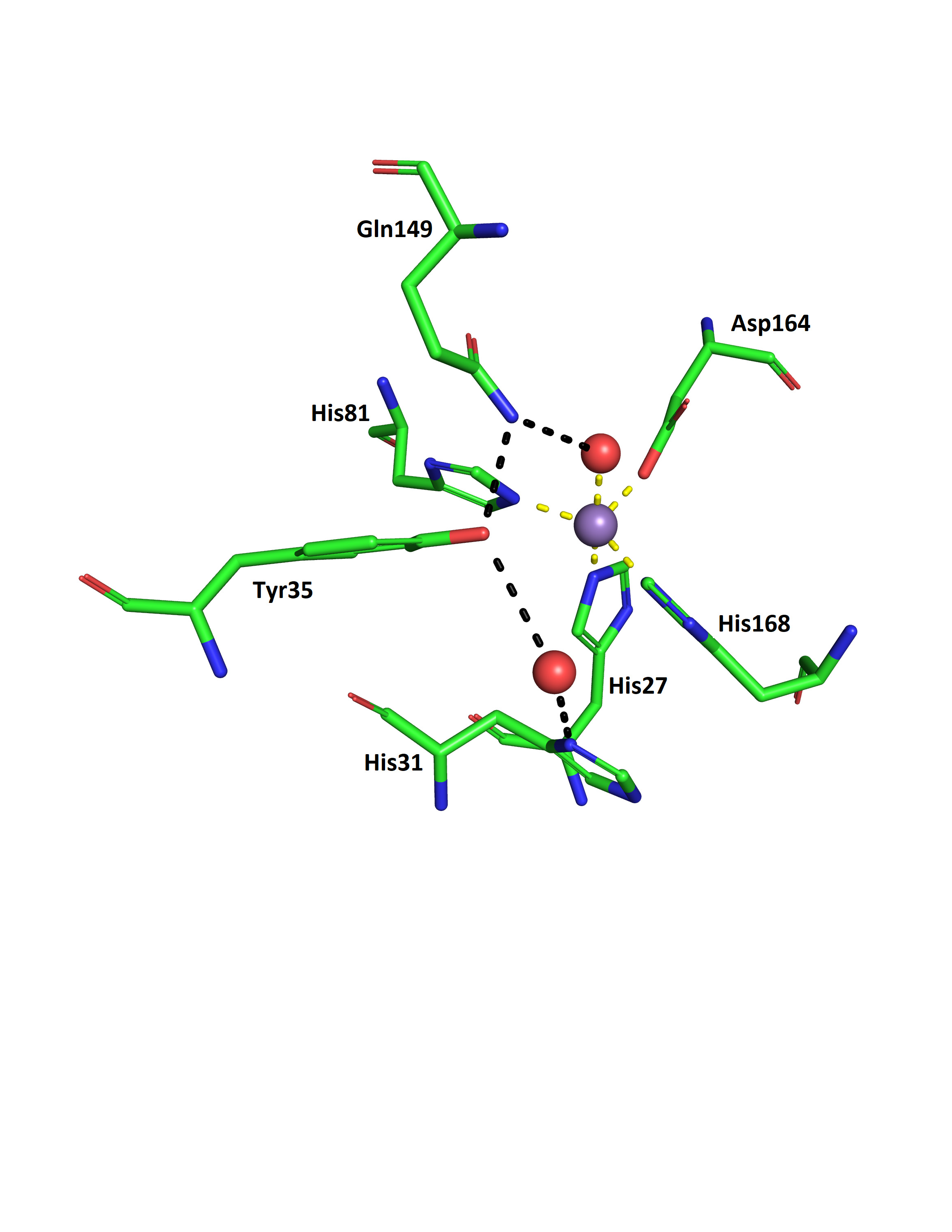

Supplement: S1 Fig — Residues around the active site which aid in securing the substrate superoxide anion are indicated. (TIFF) [file pone.0209988.s001.tiff]

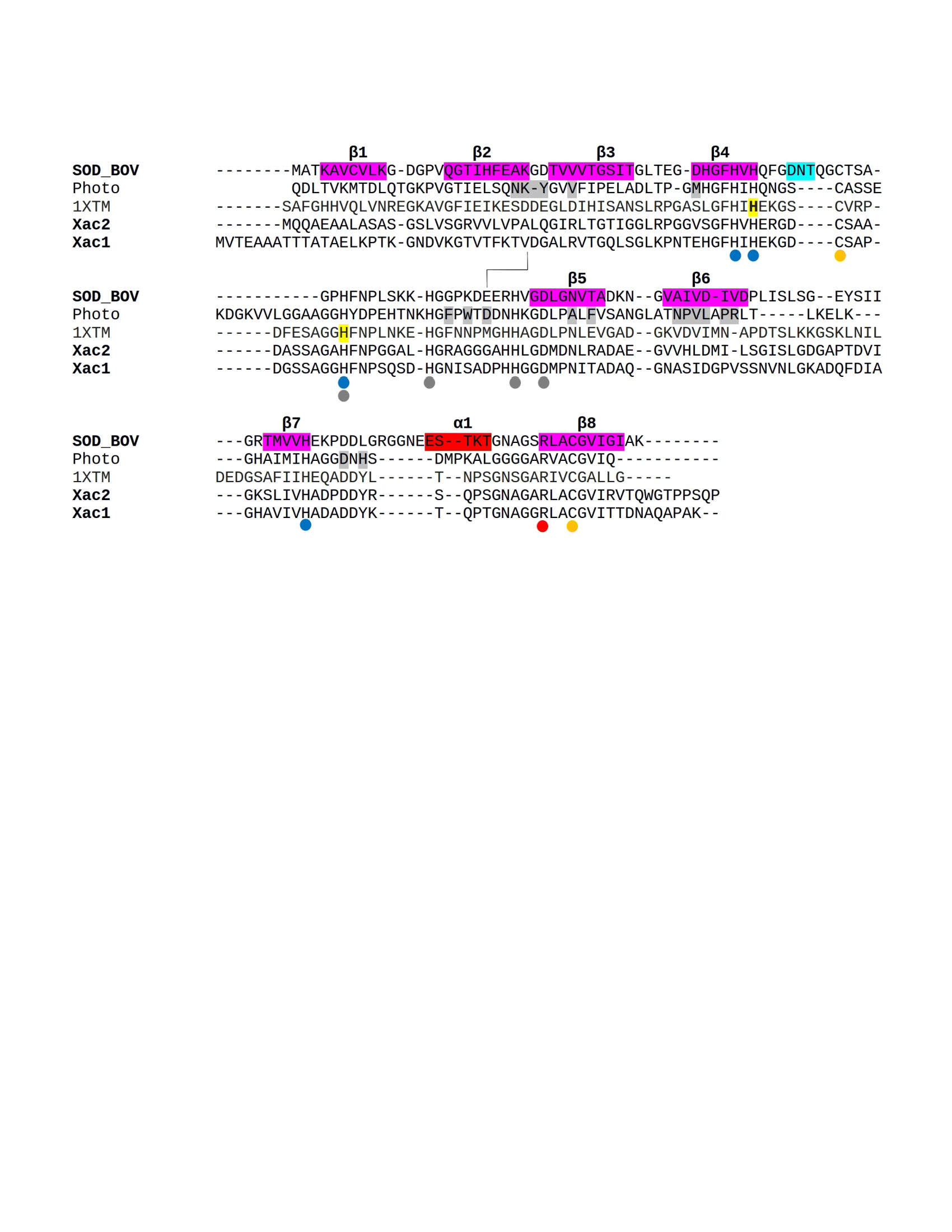

Supplement: S2 Fig — Sequence alignment of Xac1 and Xac2 was done with the bovine enzyme (SOD_BOV) and sequences from Photobacterium leiognathi (Photo), which presents an alternative dimeric interface to that seen in the bovine enzyme, and that from the SOD-like protein mutant from Bacillus subtilis (1XTM) used for model construction. Elements of secondary structure are highlighted in purple (β-strands) and red (α-helix) and the DXT motif in light blue. Residues involved in the alternative dimeric interface of the Photobacterium SOD are highlighted in grey. Copper and zinc ligands, the disulphide cysteines and the active-site arginine (important for the second half-reaction), are indicated with blue, grey, yellow and red dots respectively. (TIFF) [file pone.0209988.s002.tiff]

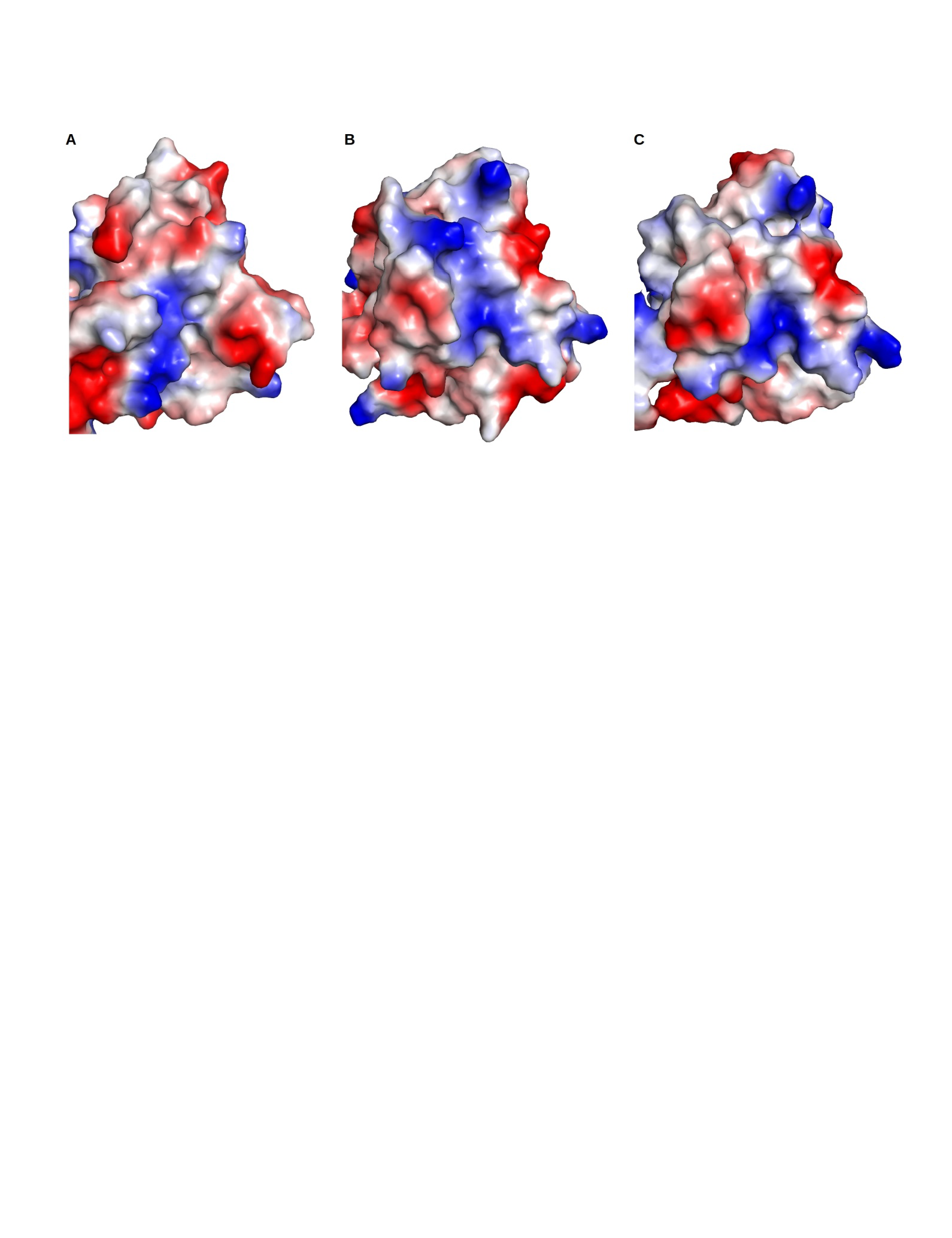

Supplement: S3 Fig — (A) shows the canonical charge distribution observed in the bovine enzyme, (B) and (C) correspond to Xac1 and Xac2 respectively. Despite their differences, all three structures show a marked positive potential leading to the active site. (TIFF) [file pone.0209988.s003.tiff]

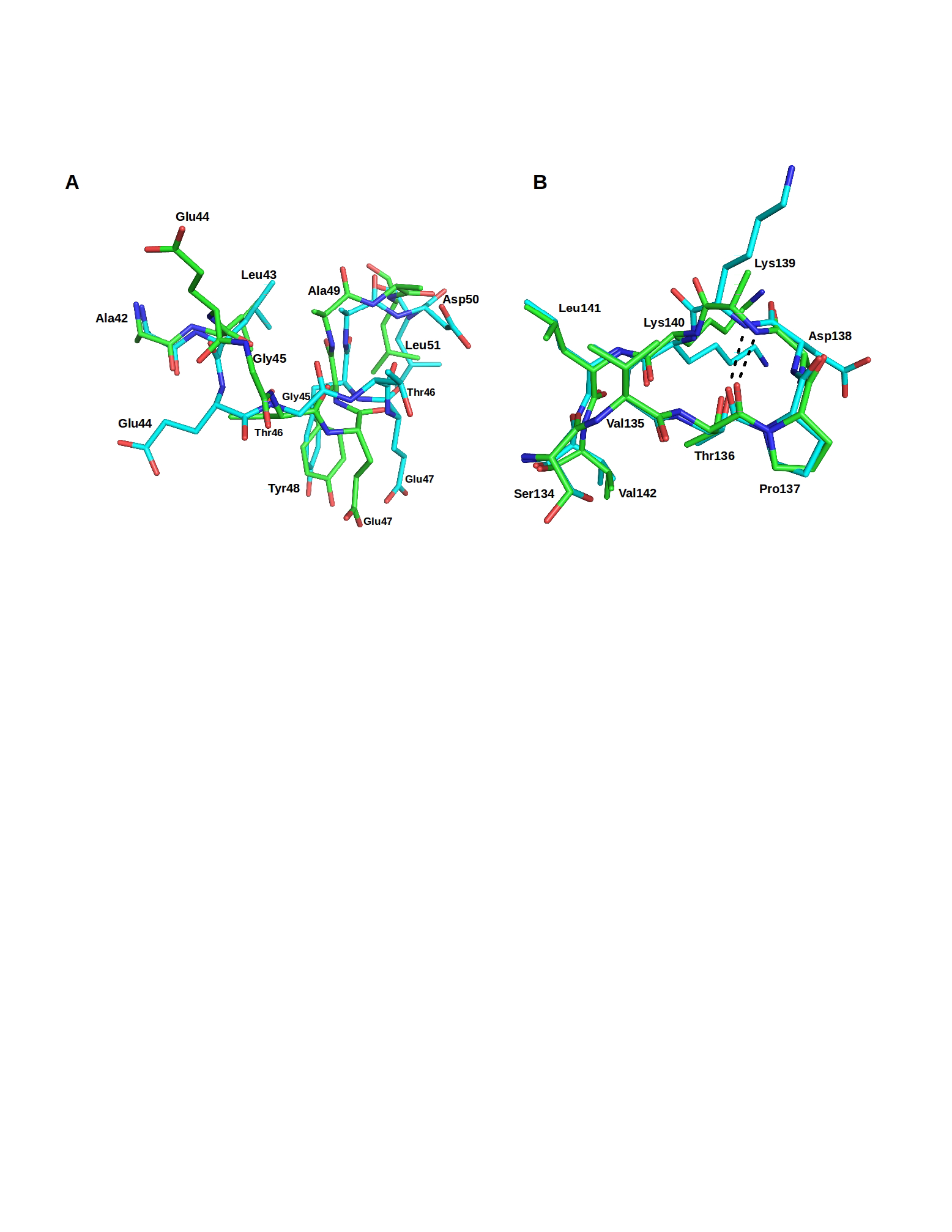

Supplement: S4 Fig — (A) shows the vicinity of residue 45 where a deletion has been incorrectly modeled by the automatic software and (B) shows the correctly predicted structure for a one-residue insertion. In both cases the crystal structure is shown in green and the homology model in blue. (TIFF) [file pone.0209988.s004.tiff]
